# Enhancement of linalool production in *Saccharomyces cerevisiae* by utilizing isopentenol utilization pathway

**DOI:** 10.1186/s12934-022-01934-x

**Published:** 2022-10-15

**Authors:** Yaoyao Zhang, Xianshuang Cao, Jin Wang, Feng Tang

**Affiliations:** Key Laboratory of National Forestry and Grassland Administration/Beijing for Bamboo & Rattan Science and Technology, International Centre for Bamboo and Rattan, No. 8 Futong Dongdajie, Wangjing, Beijing, 100102 Chaoyang District China

**Keywords:** Linalool, *Saccharomyces cerevisiae*, Metabolic engineering, Isopentenyl utilization pathway, Two-phase fermentation

## Abstract

**Background:**

Linalool is a monoterpenoid, also a vital silvichemical with commercial applications in cosmetics, flavoring ingredients, and medicines. Regulation of mevalonate (MVA) pathway metabolic flux is a common strategy to engineer *Saccharomyces cerevisiae* for efficient linalool production. However, metabolic regulation of the MVA pathway is complex and involves competition for central carbon metabolism, resulting in limited contents of target metabolites.

**Results:**

In this study, first, a truncated linalool synthase (t26AaLS1) from *Actinidia arguta* was selected for the production of linalool in *S. cerevisiae*. To simplify the complexity of the metabolic regulation of the MVA pathway and increase the flux of isopentenyl pyrophosphate (IPP) and dimethylallyl pyrophosphate (DMAPP), we introduced the two-step isopentenyl utilization pathway (IUP) into *S. cerevisiae*, which could produce large amounts of IPP/DMAPP. Further, the *S. cerevisiae IDI1* (ecoding isopentenyl diphosphate delta-isomerase) and *ERG20*^*F96W−N127W*^ (encoding farnesyl diphosphate synthase) genes were integrated into the yeast genome, combined with the strategies of copy number variation of the *t26AaLS1* and *ERG20*^*F96W−N127W*^ genes to increase the metabolic flux of the downstream IPP, as well as optimization of isoprenol and prenol concentrations, resulting in a 4.8-fold increase in the linalool titer. Eventually, under the optimization of carbon sources and Mg^2+^ addition, a maximum linalool titer of 142.88 mg/L was obtained in the two-phase extractive shake flask fermentation.

**Conclusions:**

The results show that the efficient synthesis of linalool in *S. cerevisiae* could be achieved through a two-step pathway, gene expression adjustment, and optimization of culture conditions. The study may provide a valuable reference for the other monoterpenoid production in *S. cerevisiae*.

**Supplementary Information:**

The online version contains supplementary material available at 10.1186/s12934-022-01934-x.

## Background

Terpenoids comprise the most abundant and diverse class of natural products with more than 80,000 identified compounds, widely used in medicine, flavors, biofuels and so on [1–3]. Many of those compounds have critical pharmacological properties in the treatment of cancer, malaria, inflammation, and a variety of infectious diseases [2], providing opportunities for the development of new drugs with low side effects. Linalool is an acyclic monoterpene alcohol that has a range of applications in perfumes and medicines due to its unique aroma and various biological activities such as sedative [4], antibacterial [5] and anticancer [6]. However, natural linalool, obtained mainly from spices such as camphor wood oil, is restricted by plant resources and insufficient to fulfill market demand. And synthetic linalool has poor enantioselectivity, health issues, and environmental pollution problems. Therefore, linalool synthesis in microbial platforms via fermentation has received extensive attention, which may provide a safe and sustainable alternative [7].So far, *Saccharomyces cerevisiae*, *Yarrowia lipolytica*, *Escherichia coli*, and *Pantoea ananatis* have been employed for the biosynthesis of linalool [8–11]. Nitta et al. expressed *AaLINS* from *Actinidia arguta* and the S80F mutant of farnesyl diphosphate synthase gene (*IspA*^*S80F*^) from *E. coli* combined with elevation of the precursor supply via the mevalonate pathway in *P. ananatis*, obtaining the highest ever reported linalool titer of 10.9 g/L in fed-batch fermentation [12]. *S. cerevisiae* is the attractive model organism and widely used for the commercial production of high-value chemicals due to its high safety, clear genetic background, and strong fermentation process stability [13].

Usually, the essential factors for the biosynthesis of linalool in microorganisms are linalool synthase and the immediate precursor geranyl pyrophosphate (GPP). GPP as a substrate for heterologous linalool synthase can synthesize enantioselective linalool [14]. In *S. cerevisiae*, GPP is synthesized from isopentenyl pyrophosphate (IPP) and dimethylallyl diphosphate (DMAPP) generated by the mevalonate (MVA) pathway with the catalysis of farnesyl diphosphate synthase (ERG20). However, ERG20 is a bifunctional enzyme that catalyzes the condensation of GPP and IPP into FPP, resulting in the inability to accumulate GPP. Meanwhile, FPP is an indispensable precursor in living organisms, so removing the FPP pool is not feasible. Therefore, enough GPP is one of the keys to improve linalool production. Several studies have shown that mutations at K197, F96, and N127 of ERG20 can prefer to produce GPP, especially double mutations of F96W and N127W (ERG20^F96W−N127W^) showed excellent ability to increase monoterpene production [15–17]. To date, several strategies have been established to increase the flux of GPP to linalool synthesis [8, 18–21]. A maximum linalool titer obtained in *S. cerevisiae* is 80.9 mg/L in shake-flask cultivation using a combinatorial modulation strategy that involved improving the expression level of linalool synthase by fusion a SKIK tag to N-terminus of t67OMcLIS^E343D/E352H^, increasing the conversion efficiency of GPP to linalool by attaching RIDD and RIAD peptide tags with the C-terminus of ERG20^F96W−N127W^ and SKIK-t67OMcLIS^E343D/E352H^, and downregulation of native ERG20 to reduce sterol flux [20]. Nevertheless, compared with other monoterpenes such as limonene [22, 23], the linalool production by recombinant *S. cerevisiae* is still very low.

In *S. cerevisiae*, the MVA pathway requires seven steps, two nicotinamide adenine dinucleotide phosphate (NADPH) and three Adenosine triphosphate (ATP), involving a complex regulatory mechanism and competition for central carbon metabolism, which is responsible for product yields far below the theoretical maximum. Recently, the isopentenol utilization pathway (IUP), two-step synthesis of IPP and DMAPP using isoprenol or prenol as substrates, has been developed and can replace the MVA pathway for terpenoids biosynthesis [24–29]. For instance, engineering the linalool/nerolidol synthase (bLinS) of *Streptomyces clavuligerus* in combination with the IUP to construct recombinant *E. coli* resulted in an 800-fold increase in linalool production [30]. The advantage of the IUP is that it can bypass the complexity of the inherent pathway, consume minimal cofactors, and synthesize IPP in high throughput [24, 27]. In our previous work, the linalool synthase from *Cinnamomum osmophloeum* (CoLIS) and the F96W-N127W mutant of farnesyl pyrophosphate (ERG20^F96W−N127W^) were co-expressed respectively in the mitochondria that integrated the entire MVA pathway and in the cytoplasm that expressed the key enzymes tHMG1 and IDI1 as well as down-regulated endogenous ERG20, producing 23.45 mg/L linalool by fermentation [21]. However, the growth of engineered strains was severely affected due to the metabolic burden caused by overexpression of multiple genes [21]. Therefore, we speculated the IUP introduced into *S. cerevisiae* can simplify metabolic regulation and reduce the metabolic burden.

**Fig. 1:**
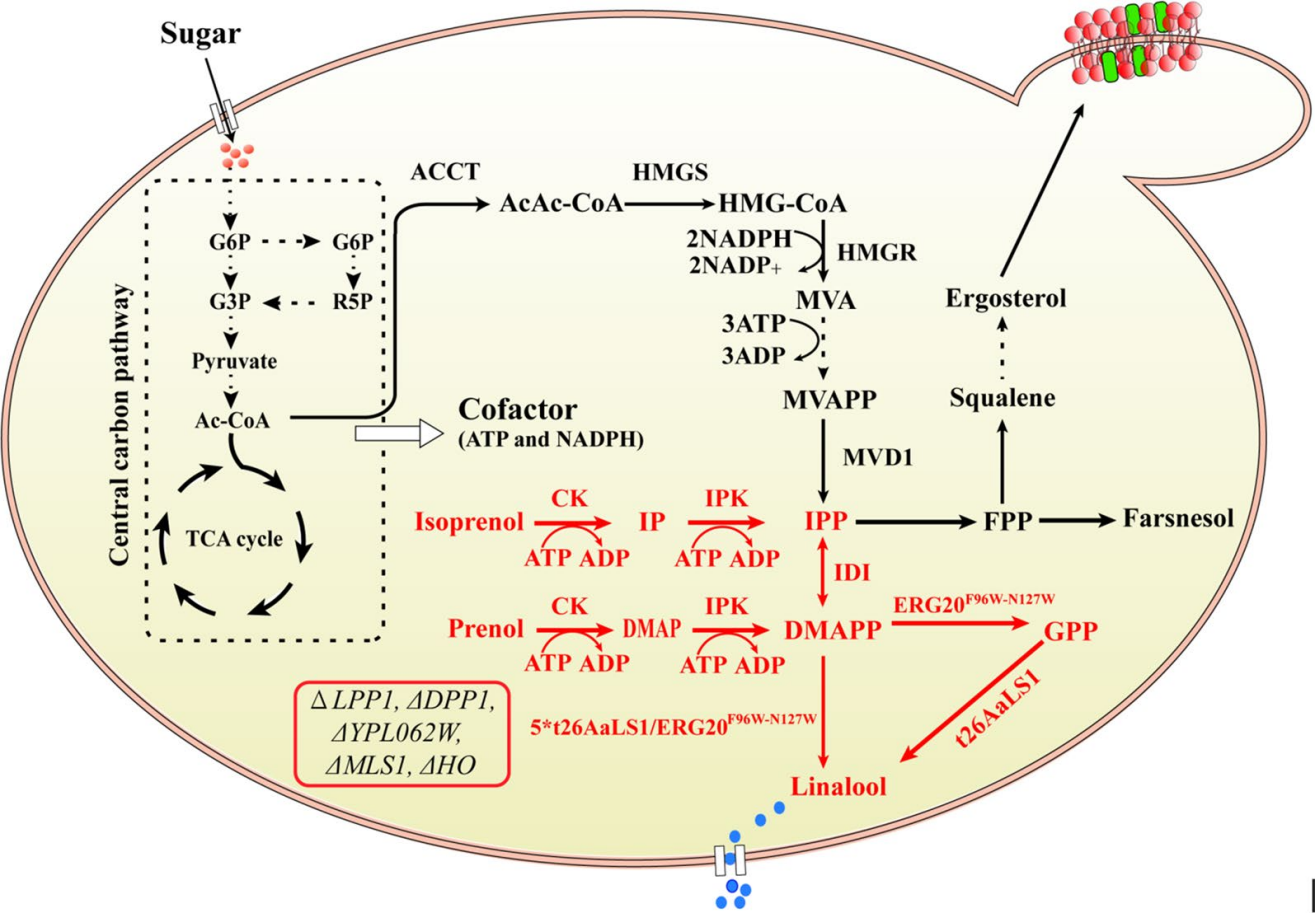
Scheme for linalool production in *S. cerevisiae* using the IUP. Native pathways in *S. cerevisiae* are shown by the black arrows. Engineered linalool biosynthesis pathway is shown by the red arrows. The insertion sites of five copies of *t26AaLS1* and *ERG20*^*F96W−N127W*^ genes on the yeast genome are shown in the red box. The enzymes involved in the linalool biosynthesis pathway as follows: ScCK, choline kinase from *S. cerevisiae* S288C; AtIPK, isopentenyl phosphate kinase from *A. thaliana*; IDI1, isopentenyl diphosphate delta-isomerase from *S. cerevisiae* S288C; ERG20^F96W−N127W^, geranyl pyrophosphate synthase from *S. cerevisiae* S288C; t26AaLS1, linalool synthase from *A. arguta*

In the work, we sought to utilize the IUP to boost the production of linalool in *S. cerevisiae* (Fig. 1). First, different N-terminal truncated linalool synthases from *Actinidia arguta* were individually overexpressed in engineered *S. cerevisiae* to investigate the effect on linalool production in *S. cerevisiae*. Subsequently, the IUP was introduced into *S. cerevisiae*, and intracellular IPP and DMAPP contents were compared using the engineered MVA pathway and the IUP to determine the utility of IUP in *S. cerevisiae*. Further, three key enzyme genes involved in the linalool biosynthesis pathway were integrated into the *S. cerevisiae* genome and meanwhile optimized isoprenol and prenol to increase the linalool production. In addition, the effect of *t26AaLS1* and *ERG20*^*F96W−N127W*^ genes copy number on the linalool production was also examined. Finally, through the optimization of carbon source and Mg^2+^, the linalool production was increased to a high level in engineered *S. cerevisiae*.

## Results and discussion

### Comparison of two linalool synthases for linalool production in engineered *S. cerevisiae*

**Fig. 2:**
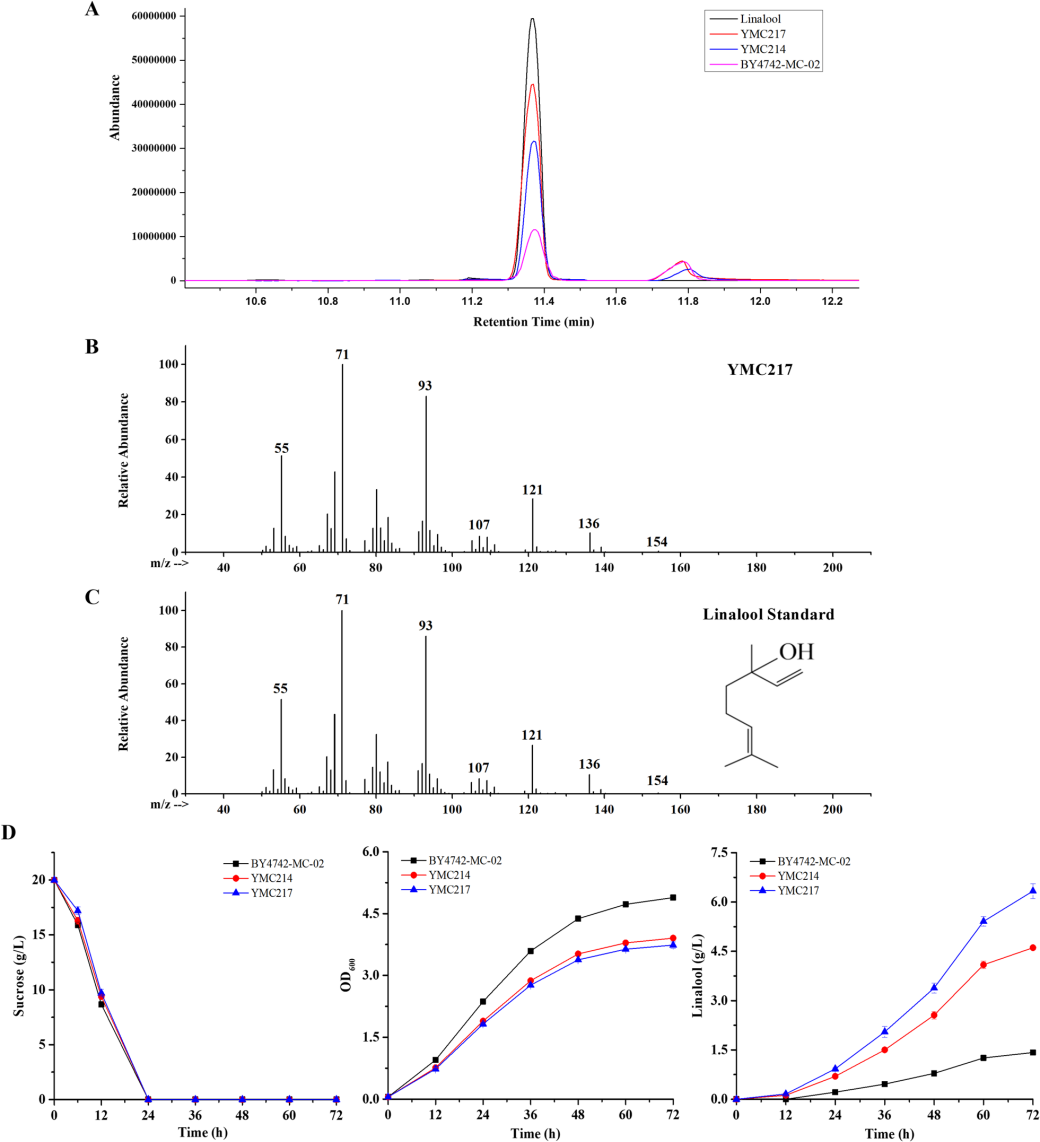
Linalool production from engineered *S. cerevisiae* strains. **A** Total ion chromatogram for linalool standard, the engineered strain YMC214, YMC217 and the control strainBY4742-MC-02. **B** Mass spectra for linalool production by the strain YMC217. **C** Mass spectra for linalool standard. **D** Sucrose consumption, cell growth and linalool production curves of the engineered strain YMC214, YMC217 and the control strain BY4742-MC-02. The strains were cultured in SS-URA medium with 20% isopropyl myristate (IPM) for 72 h

Generally, plant-derived linalool synthases exhibit low catalytic efficiency when heterologously expressed and are often considered the impediment to high linalool production. In a previous study, the performance of six (*S*)-specific linalool synthases from the plants was compared in *Pantoea ananatis*, and *Actinidia arguta* linalool synthase (AaLS1) showed the best (*S*)-linalool production with 100% enantio excess [31]. Therefore, to optimize heterologous linalool production in *S. cerevisiae*, the co-expression plasmid pYC-*MAaLS1*-*MERG20*^*F96W−N127W*^ including *AaLS1* and *ERG20*^*F96W−N127W*^ with mitochondrial localization signal (MLS) sequence was transformed into strain BY4742-MC-02, generating strain YMC217. *S. cerevisiae* BY4742-MC-02 was reported in our previous work [21], which showed the strain with the whole MVA pathway in both mitochondria and cytoplasm and with a sufficient precursor supply. The strain YMC214 harboring the pYC-*MLIS*-*MERG20*^*F96W−N127W*^ plasmid including (*S*)-linalool synthase gene from *Cinnamomum osmophloeum* was used as the experimental group and BY4742-MC-02 was used as the control. Since the parental strain BY4742-MC-02 has a *GAL80* deletion, genes under the control of the GAL promoter also allows gene expression in the absence of galactose. Thus, GC-MS analysis was performed after the recombinant strains were cultured in SS-URA medium with 20% isopropyl myristate (IPM) for 72 h. As shown in Fig. 2, linalool was detected in YMC217, and the retention time was 11.37 min. Specifically, although both the strains YMC217 and YMC214 had little difference in the cell growth and sucrose consumption ability (Fig. 2D), the production of linalool by YMC217 overexpressing *AaLS1* was determined to be 12.67 mg/L and showed 1.4-fold higher than that by YMC214 overexpressing *CoLINS* (9.22 mg/L). The results reflected that AaLS1 could at least equally or more effectively convert GPP to linalool than CoLINS used in our previous work [21].

**Fig. 3:**
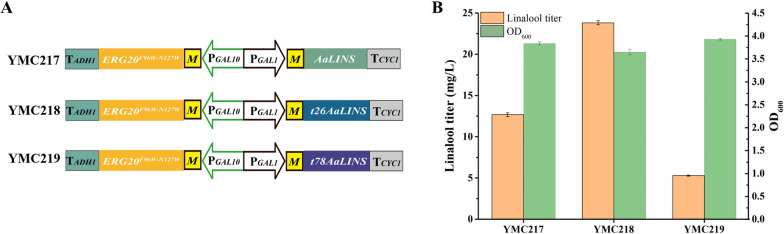
Effects of different truncation position in N-terminus of *AaLS1* and IPM layer on linalool production. **A** Scheme of the screening strategy for the truncated position of N-terminus of *AaLS1*. **B** Linalool production and cell growth in strains overexpressing *AaLS1*, *t26AaLS1* and *t78AaLS1*, respectively. The strains were cultured in SS-URA medium with 20% isopropyl myristate (IPM) for 72 h. All values represent the mean ± standard deviation from three biological replicates

Meanwhile, monoterpene synthases in plants have an N-terminal plastidic transit peptide, and the proper truncation site for monoterpene synthases can improve microbial monoterpene production [31]. The RR-heuristic motif is a conserved double arginine motif and residues upstream of the highly conserved RR motif are not required for monoterpene synthase activity [32]. And a previous study reported that deletion of residues upstream of the RR motif improved monoterpene synthase expression in yeast [31]. Thus, to improve the expression and activity of linalool synthase in *S. cerevisiae*, the N-terminal plastid targeting sequence truncation site of *AaLS1* was predicted by the ChloroP or RR-heuristic method to obtain t26AaLS1 and t78AaLS1. The *t26AaLS1* and *t78AaLS1* fragments were obtained to construct pYC-*Mt26AaLS1*-*MERG20*^*F96W−N127W*^ and pYC-*Mt78AaLS1*-*MERG20*^*F96W−N127W*^, respectively, which were transformed into strain BY4742-MC-02, generating the strains YMC218 and YMC219, respectively (Fig. 3A). The results showed that YMC218 carrying *t26AaLS1* produced 23.81 mg/L of linalool, which was almost 1.9-fold higher than that of YMC217 carrying *AaLS1* (Fig. 3B). Nevertheless, YMC219 carrying *t78AaLS1* produced 5.29 mg/L of linalool and did not increase the production of linalool but harmed linalool synthesis (Fig. 3B), most likely due to improper N-terminal truncation position that impaired the activity of t78AaLS1. Similarly, the highest linalool titer and geraniol titer in *S. cerevisiae* from truncated *Actinidia polygama* LIS (t26ApLIS) and truncated *Phyla dulcis* geraniol synthase (t43PdGES), respectively, while the *S. cerevisiae* strains expressing t78ApLIS and t86PdGES produced less linalool and geraniol than ApLIS and PdLIS [31]. In addition, an aqueous-organic two-phase system to extract fermentation of terpenoids in recombinant *S. cerevisiae* is adopted as an effective strategy to reduce product toxicity and collect hydrophobic products [33, 34]. Isopropyl myristate (IPM) is a commonly used solvent in two-phase extractive fermentation and has been used to improve the production of terpenoids such as Alpha-Terpineol and linalool [17, 19, 20, 23]. A recent study showed that the use of isopropyl myristate as the extraction solvent in the ultrasonic-irradiated two-phase extractive fermentation significantly improved the carotenoid production of recombinant *S. cerevisiae*, and isopropyl myristate exhibited obvious advantages over dodecane and butyl oleate in the extraction of carotenoids [35]. To evaluate the effects of linalool production in two-phase extract fermentation using IPM, the above engineering strains in this study were cultured in SS-URA medium with and without IPM. When 20% IPM was added to the culture medium, the yield of linalool by YMC218 reached 23.94 mg/g DCW, representing a 103% increase compared with no overlay (11.81 mg/g DCW). As shown during linalool production, we confirmed that the medium covered with IPM can improve the production of linalool and hardly affect the growth of the strain. Furthermore, we also tested the effect of different concentrations of linalool added to the SS-URA medium on growth of the engineered *S. cerevisiae* strain. The results showed that the cell growth was significantly inhibited by more than 250 mg/L linalool (Additional file 1: Fig. S1).

### Effects of introducing the IUP on synthesis of IPP/DMAPP in *S. cerevisiae*

Before constructing IUP in *S. cerevisiae*, we examined the tolerance of *S. cerevisiae* against isoprenol and/or prenol due to their toxicity to microbial hosts, affecting cell growth and target product yield [36, 37]. *S. cerevisiae* strain BY4742 was cultured in YPD medium supplemented with isoprenol and/or prenol at different concentrations. And optical density at 600 nm (OD_600_) was taken to evaluate the toxicity of isoprene and prenol in *S. cerevisiae*. After 24 h, the cell growth decreased by 55% and 73% for the addition of 100 mM isoprenol or prenol, respectively, compared with that without isoprenol or prenol (Additional file 1: Fig. S2A, B). With the addition of 200 mM isoprenol or prenol, cell growth was severely inhibited. A previous study has also shown that *S. cerevisiae* was more tolerant to isoprenol than *E. coli*, with a maximum half-inhibitory concentration of 7.79 g/L (~ 90 mM) to isoprenol, and *S. cerevisiae* showed no growth at 20 g/L isoprenol (~ 232 mM) [37]. When supplemented with 50 mM isoprenol and prenol, biomass decreased by 65%, and cell growth was dramatically affected (Additional file 1: Fig. S2C).

**Fig. 4:**
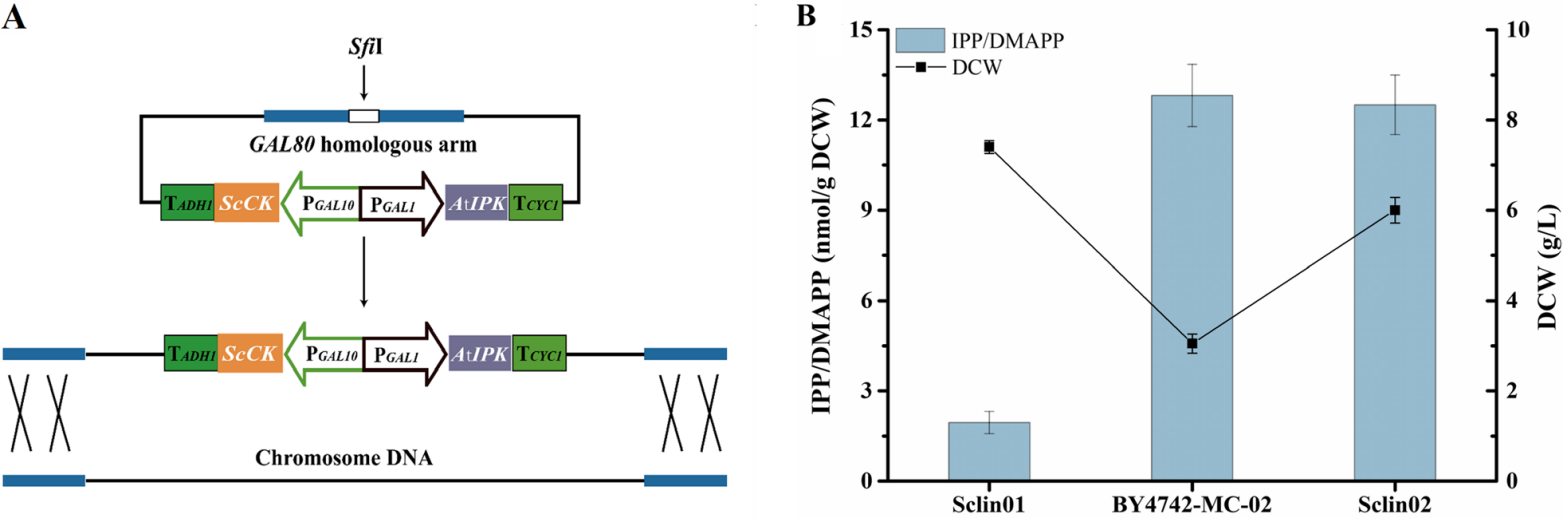
Effect of introducing the IUP on the synthesis of IPP and DMAPP. **A** Scheme of introducing the IUP into *S. cerevisiae*. **B** Comparison of intracellular IPP and DMAPP contents in *S. cerevisiae* control strain Sclin01, strain BY4742-MC-02 with the whole MVA pathway in both mitochondria and cytoplasm, and strain Sclin02 with the IUP. Strains Sclin01 and Sclin02 were cultured in YPS medium containing 17.5 mM isoprenol and prenol, respectively, and BY4742-MC-02 was grown in YPS medium

Although in our previous study, BY4742-MC-02 we constructed could increase the production of linalool through dual regulation of MVA pathways in the mitochondrial and cytoplasmic, the biomass was significantly reduced due to the excessive metabolic burden [21]. Several studies have demonstrated that the IUP can avoid complex regulation and central carbon metabolism, achieving efficient biosynthesis of terpenoids [24–29]. Therefore, to simplify the means of metabolic regulation and reduce the metabolic burden, we introduced IUP into *S. cerevisiae*. In order to use glucose or sucrose as a carbon source to regulate the expression of genes in the system, Sclin01 was obtained by knocking out the *GAL80* gene on the BY4742 genome, which was the control group. It was reported that choline kinase from *S. cerevisiae* (ScCK) could convert isoprenol to appreciable amounts of both IP and DMAP and isopentenyl phosphate kinase from *Arabidopsis thaliana* (AtIPK) exhibited excellent performance in catalyzing the second step of IUP [24]. Thus, the *AtIPK* and *ScCK* genes encoding the IUP were cloned in the pUMRI-G plasmid, resulting in the plasmid pUMRI-G-*AtIPK*-*ScCK*. This plasmid was transformed into BY4742 so that the *AtIPK* and *ScCK* genes were inserted into the GAL80 locus in strain BY4742, generating strain Sclin02 (Fig. 4A). Subsequently, the intracellular IPP/DMAPP contents were analyzed by LC-MS/MS. As expected, a small amount of intracellular IPP/DMAPP was detected in Sclin01 using the native MVA pathway as a control, while the IUP-introduced strain Sclin02 significantly increased by 6.4-fold. And the biomass of Sclin02 decreased by 18.91% compared to Sclin01 (Fig. 4B). Importantly, IPP/DMAPP contents were not significantly different between Sclin02 and BY4742-MC-02, but the biomass of Sclin02 was 2.0-fold higher than that of BY4742-MC-02 (Fig. 4B). The effects of introducing IUP into *S. cerevisiae* on the synthesis of IPP and DMAPP in our study were consistent with that of introducing IUP into the peroxisome of *Pichia pastoris* (i.e., 7.8-fold) [38], but neither exhibited excellent performance as the introduction of IUP into the cytoplasm of *Yarrowia lipolytica* (i.e., 15.7-fold) [28]. The phenomenon may be related to the limited permeability of cells [38]. Overall, the results indicate that the IUP is an efficient strategy for the two-step synthesis of native MVA pathway products IPP/DMAPP in *S. cerevisiae*.

### Enhancement linalool synthesis in *S. cerevisiae* using the IUP

To evaluate the utility of this strategy on linalool production in *S. cerevisiae*, *t26AaLS1* and *ERG20*^*F96W−N127W*^ were first introduced into Sclin02 by the plasmid pUMRI-H-*t26AaLS1-ERG20*^*F96W−N127W*^, generating the strain Sclin03. Meanwhile, previous studies have shown that it is an effective strategy to increase the synthesis of terpenoids by either adding an appropriate concentration of isoprenol [28] or controlling the ratio between isoprenol and prenol [26, 30]. Thus, Sclin03 was first tested for linalool production using various concentrations of isoprenol and/or prenol. After 96 h two-phase shake-flask culture, a maximum linalool titer of 23.44 mg/L supplied with 17.5 mM each of isoprenol and prenol was obtained. And linalool was also detected supplemented with other concentrations of isoprenol and prenol in combination (data not shown). However, no linalool was detected in cultures supplemented with only one of the two, probably due to isoprenol and prenol producing separately large amounts of IPP and DMAPP through the IUP pathway, resulting in excessive content of one of them not conducive to the formation of linalool.

*IDI1* and *ERG20* are the key enzyme genes in the linalool synthesis pathway, which play individually important roles in the interconversion and balance of IPP and DMAPP and the synthesis of GPP available, affecting the linalool production [39]. Thus, to investigate the effects of *IDI1* and *ERG20*^*F96W−N127W*^ on the production of linalool based on the IUP, two copies of *IDI1*, one copy of *IDI1* and *ERG20*^*F96W−N127W*^ were introduced into Sclin03, respectively, generating Sclin04 and Sclin05. As expected, the results showed that overexpression of either two copies of *IDI1* or one copy of *IDI1* together with *ERG20*^*F96W−N127W*^ increased linalool production. Different contents of linalool were produced in Sclin04 when various concentrations of prenol were added to the medium (data not shown). However, with the addition of isoprenol to the medium, nerolidol significantly increased while linalool was not detected. We speculate that the conversion of IPP to DMAPP catalyzed by IDI1 enzyme is restricted, resulting in the unbalanced ratio of IPP and DMAPP, while the precursor GPP for linalool synthesis requires a 1:1 ratio of IPP to DMAPP, and when a lower intracellular level of DMAPP than IPP, IPP may still react with GPP to form other terpenoid precursors, such as FPP, resulting in a decrease in the metabolic flux of GPP to linalool. Notably, the highest titer of linalool in strain Sclin04 represented approximately 1.6-fold increases relative to Sclin03 at 17.5 mM each of isoprenol and prenol, reaching about 37.01 mg/L. To sum up, the results revealed that the combination of isoprenol and prenol had a significant effect on linalool production, which was similar to the results obtained in previous studies [25, 30].

**Fig. 5:**
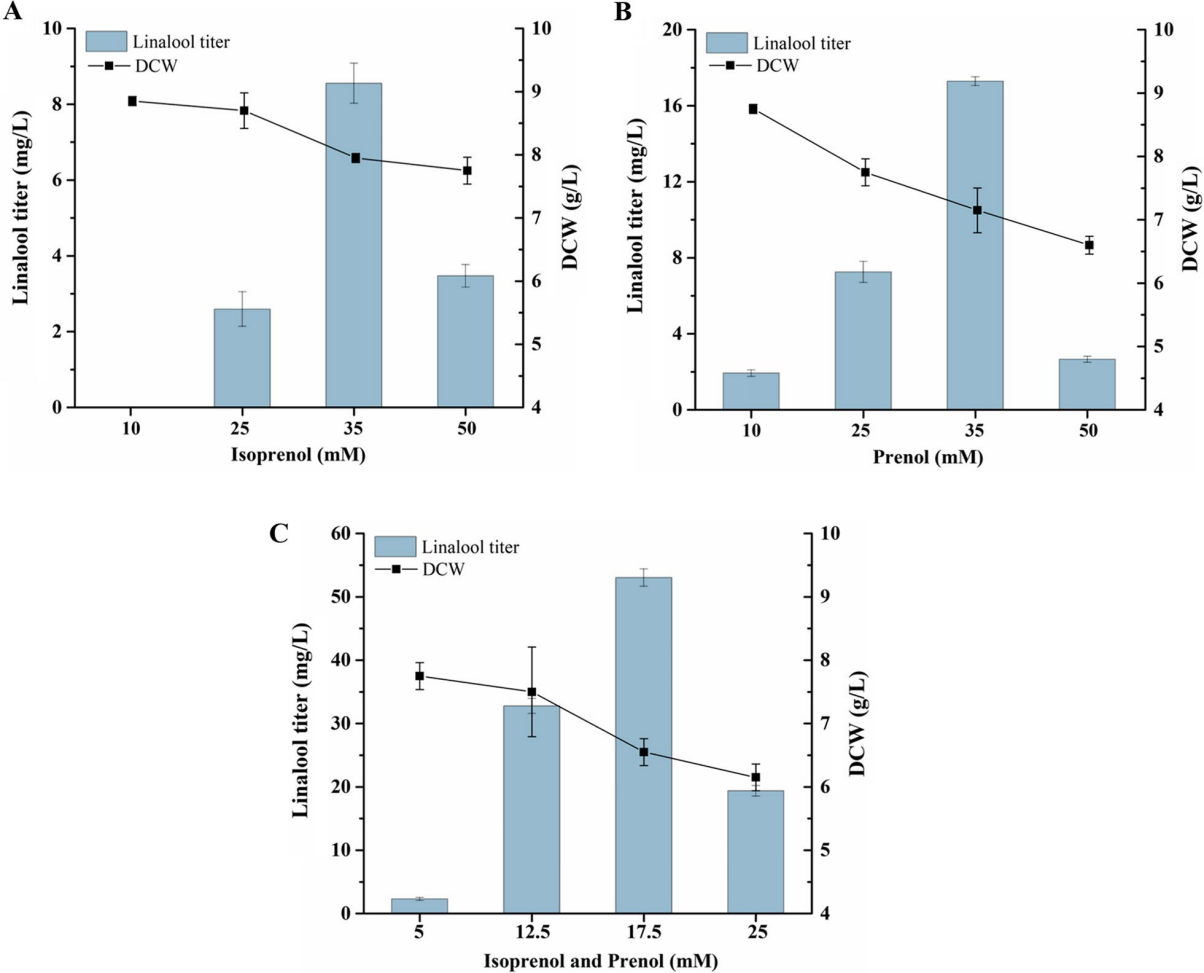
Effects of different combinations of isoprenol and prenol on linalool production. **A** Isoprenol was added to the YPS medium of strain Sclin05 to a final concentration of 10, 25, 35, and 50 mM. **B** Prenol was added to the YPS medium of strain Sclin05 to a final concentration of 10, 25, 35, and 50 mM. **C** Isoprenol and prenol were together added to the YPS medium of strain Sclin05 to a final concentration of 5, 12.5, 17.5, and 25 mM. The experiments were covered with 20% IPM and incubated in shake flasks for 72 h. All values represent the mean ± standard deviation from three biological replicates

When Sclin05 was tested using various concentrations of isoprenol and/or prenol, the results showed that the strain produced 8.55 mg/L linalool at 35 mM isoprenol, 17.28 mg/L at 35 mM prenol, and 53.03 mg/L at 17.5 mM each of isoprenol and prenol (Fig. 5). Specifically, the highest linalool titer in Sclin05 was almost 1.4-fold higher than that in Sclin04 under the same conditions, indicating that overexpression of *IDI1* and *ERG20*^*F96W−N127W*^ could better improve the production of linalool. The results illustrate that overexpression of *IDI* and *ERG20* can enhance the downstream metabolic synthesis flux of IPP [28, 29]. In addition, in order to fully utilize IPP and DMAPP to convert into GPP, the geranyl pyrophosphate synthase gene from *Abies grandis* with the plastid-targeting sequence removed to generate *tAgGPPS*, which was inserted into strain Sclin03 together with *IDI1*. Unexpectedly, the resulting strain Sclin06 produced 24.65 mg/L of linalool, which decreased by 53% compared with Sclin05, although a previous study showed that overexpression of *AgGPPS* increased the production of geraniol in *S. cerevisiae* with *ERG20*^*K197E*^ [40]. Herein, plant-derived GPP synthases introduced into *S. cerevisiae* to promote monoterpene synthesis requires further investigation.

### Effect of increasing gene copy numbers on linalool production

**Fig. 6:**
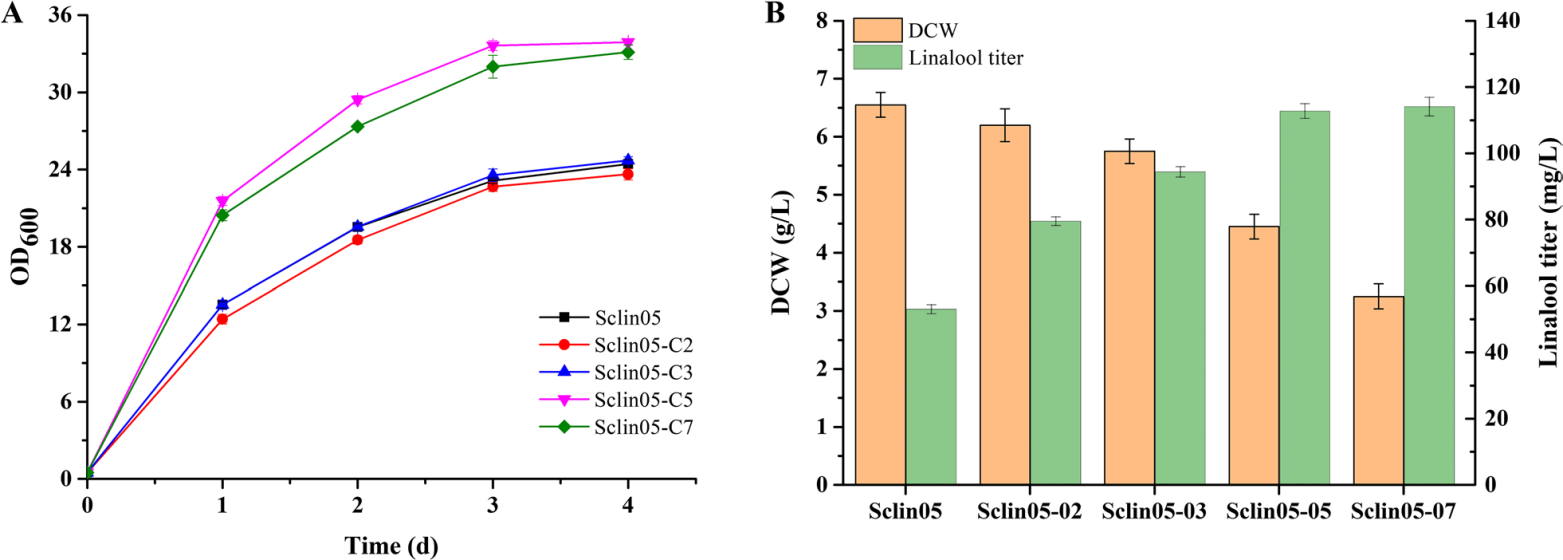
Linalool production by chromosomally engineered *S. cerevisiae* Sclin05. **A** Cell growth curves of the engineered strain deleted off-pathway genes. **B** Effects of enhancing gene expression on linalool production. The strains were cultured in YPS medium supplemented with 17.5 mM isoprenol and prenol and with 20% IPM for three or four days. All values represent the mean ± standard deviation from three biological replicates

The use of IUP to produce terpenoids may have to deal with the low utilization of IPP and DMAPP by downstream terpenoid synthesis [24]. Enhancing gene expression by regulating gene copy number is an effective approach to increasing metabolic pathway flux [29, 41]. For example, increasing the copy number of the related rate-limiting enzymes genes *CrtE* (encoding geranylgeranyl pyrophosphate synthase) and *CrtI* (encoding phytoene desaturase) in *S. cerevisiae* increased lycopene production from 118 mg/L to 186 mg/L [41]. As such, to further improve the production of linalool, 2, 3, 5, and 7 copies of *t26AaLS1* and *ERG20*^*F96W−N127W*^ were introduced into the genome of Sclin05, generating the strains Sclin05-2, Sclin05-3, Sclin05-5, and Sclin05-7, respectively, which required searching for suitable insertion sites on the genome. Genes *LPP1* and *DPP1* encode two phosphatases that accounted for most of the hydrolytic activities and deleting genes *LPP1* and *DPP1* could improve the production of terpenoids in yeast [42]. We first explored the effect of deleting *LPP1* and *DPP1* on cell growth and linalool production in strain Sclin05. The *LPP1* and *DPP1* genes were knocked out in Sclin05, generating single-knockout Sclin05-C2 (Δ*LPP1*) and double-knockout Sclin05-C3 (Δ*LPP1,* Δ*DPP1*), respectively. The results showed no visible effect on cell growth (Fig. 6A) and linalool production (data not shown) compared with Sclin05. Subsequently, *t26AaLS1* was inserted together with *ERG20*^*F96W−N127W*^ into the *LPP1* or *LPP1* and *DPP1* sites in Sclin05 using the plasmids pUMRI-L-*t26AaLS1*-*ERG20*^*F96W−N127W*^ and pUMRI-D-*t26AaLS1*-*ERG20*^*F96W−N127W*^, generating strain Sclin05-2 and Sclin05-3, respectively. As shown in Fig. 6B, the linalool titer of Sclin05-2 and Sclin05-3 was 79.48 mg/L and 94.39 mg/L, which were 49.9% and 78.0% higher than that of Sclin05, respectively. To investigate whether the genes *t26AaLS1* and *ERG20*^*F96W−N127W*^ were still the rate-limiting steps for Sclin05-3, we constructed Sclin05-5 and Sclin05-7 expressing 5 and 7 copies of *t26AaLS1* together with *ERG20*^*F96W−N127W*^, respectively. The *MLS1* gene*,* encoding cytosolic malate synthase, is involved in acetyl-CoA catabolism, and the deletion of the *MLS1* gene can reduce the competition for acetyl-CoA with the MVA pathway and increase the production of terpenoids [23, 43]. And several studies have reported that deleting genes *YPL062W*, *YJL064W*, and *ROX1* can improve the production of terpenoids in yeast [23, 29, 44, 45]. Thus, we further explored the effect of deleting genes *MLS1*, *YPL062W*, *YJL064W*, and *ROX1* on cell growth and linalool production in Sclin05-C3. The above genes were knocked out in Sclin05-C3, generating double-knockout Sclin05-C5 (Δ*MLS1,* Δ*YPL062W*) and quadruple-knockout Sclin05-C7 (Δ*MLS1,* Δ*YPL062W,* Δ*YJL064W,* Δ*ROX1*), respectively, resulting in a 37% increase in biomass (Fig. 6A) but no visible effect on linalool production (data not shown). Similarly, *t26AaLS1* together with *ERG20*^*F96W−N127W*^ were inserted into *MLS1*, *YPL062W*, *YJL064W*, and *ROX1* sites in Sclin05-3, generating Sclin05-5 and Sclin05-7, respectively. Compared with the linalool titer in Sclin05-3, the titer of linalool was increased by 19.4% to 112.75 mg/L in Sclin05-5 and 20.8% to 114.08 mg/L in Sclin05-7, respectively (Fig. 6B). The results indicated that further integration of another two or four copies of *t26AaLS1* together with *ERG20*^*F96W−N127W*^ only led to a marginal increase in linalool production of the strains Sclin05-5 and Sclin05-7 when compared with Sclin05-3, and there was no significant change in linalool titer between Sclin05-5 and Sclin05-7. Moreover, compared to Sclin05-5, the biomass of Sclin05-7 decreased by 27%. Similarly, a previous study showed that adding one copy of *CrtI* increased lycopene production by 80.6% while introducing three copies of *CrtI* decreased both lycopene production and biomass [46]. The reason may be that excessive copy number increase imposes a metabolic burden on microbial growth [47]. Finally, the linalool production reached the maximum in the engineered strain Sclin05-5. The data suggested that increased downstream terpenoid metabolic flux is necessary when utilizing IUP to increase the heterologous synthesis of terpenoids.

### Effects of carbon sources and Mg^2+^ addition in medium

**Fig. 7:**
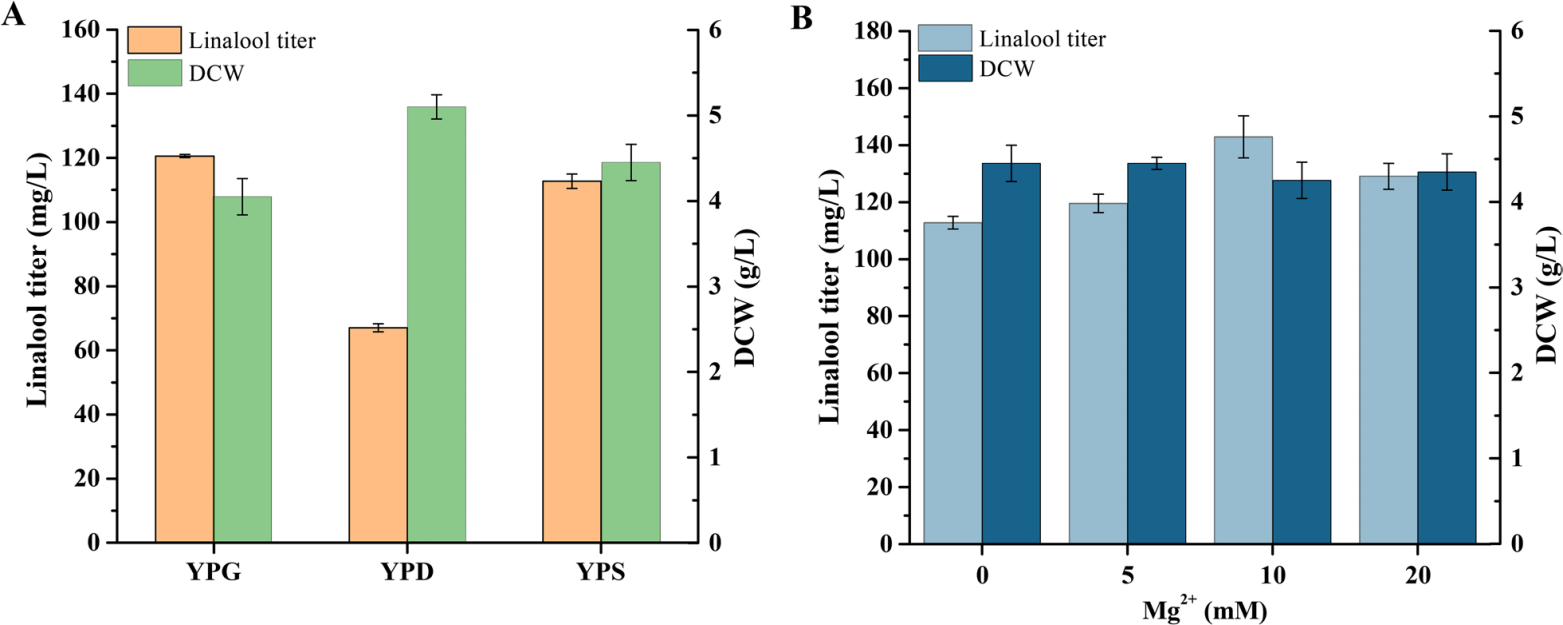
Effects of different carbon sources and Mg^2+^ addition on linalool production of strain Sclin05-5. **A** 20 g/L glucose, galactose, and sucrose respectively as the sole carbon source in YP medium. **B** Mg^2+^ was added to the YPS medium to a final concentration of 0, 5, 10, or 20 mM. The strains were cultured in corresponding medium supplemented with 17.5 mM isoprenol and prenol and with 20% IPM for 72 h. All values represent the mean ± standard deviation from three biological replicates

Carbon sources can directly influence microbial growth and provide energy and carbon skeletons for the reproduction of microorganisms via a series of chemical reactions in vivo. In the strain Sclin05-5, the *GAL* regulation system was modified by *GAL80* deletion, resulting in independence of gene expression on the absence of galactose. Depletion of glucose was able to induce the expression of P_*GAL*_-driven genes in this background [48, 49]. Therefore, glucose, galactose and sucrose were used individually as the carbon source of strain Sclin05-5 to explore the effect of carbon sources on the production of linalool. As shown in Fig. 7A, when galactose and sucrose were added to the medium separately to provide energy and carbon skeleton for the reproduction of microorganisms, the linalool yield reached 120.92 mg/L and 112.75 mg/L after being cultivated for 96 h, indicating that strain Sclin05-5 could utilize sucrose to produce linalool. And there was no significant difference in cell growth. The lowest linalool titer (43.58 ± 1.04 μg/L) was detected in YPD medium, although the cells grew better in YPD medium than in YPG and YPS.

Generally, Mg^2+^ plays a role in the process of growth and metabolism of living cells [50] and is related to the activity of monoterpene synthase [51, 52]. A previous study showed that adding Mg^2+^ to the medium increased the production of limonene by engineered *Yarrowia lipolytica* [52]. Herein, the effect of different concentrations of Mg^2+^ added to the medium on the linalool production by engineered *S. cerevisiae* was investigated. No significant changes were found in DCW under the experimental conditions, indicating that adding of Mg^2+^ had no visible effect on the growth of the engineered *S. cerevisiae* (Fig. 7B). With the addition of 10 mM Mg^2+^ to the medium, a maximum titer of linalool reached 142.88 mg/L, 27% higher than the control group (Fig. 7B). The results show that the optimized Mg^2+^ concentration can further improve the linalool titer.

## Conclusion

In the present work, a *S. cerevisiae* platform was successfully developed as a high-linalool producer using IUP, a heterologously expressed linalool synthase, and an ERG20 mutation in *S. cerevisiae* to increase the linalool synthesis in *S. cerevisiae*. We first selected the truncated *Actinidia arguta* linalool synthase t26AaLS1, which improved linalool production when overexpressed in engineered *S. cerevisiae* strain with dual regulation of the MVA pathway in the mitochondria and cytoplasm, obtaining a linalool titer of 23.81 mg/L, a 2.6-fold increase compared to CoLIS. Further, by integrating *t26AaLS1*, *ERG20*^*F96W−N127W*^, and *IDI* into the yeast genome, the linalool titer in Sclin05 was 53.03 mg/L, a 2.3-fold increase compared to Sclin03, demonstrating the potential of utilizing the IUP pathway to increase linalool synthesis. Additionally, the strategy of copy number variation of the *t26AaLS1* and *ERG20*^*F96W−N127W*^ genes was employed to increase the metabolic flux of the downstream IPP, resulting in a significant increase in the linalool titer. Finally, strain Sclin05-5 was grown in YPS medium supplemented with 10 mM Mg^2+^, 17.5 mM isoprenol and prenol, and a maximum linalool titer reached 142.88 mg/L in a shake flask, which was the highest reported yet in engineered yeast strains, demonstrating the utility of these strategies for monoterpenes biosynthesis in yeast. Although we also tried to use plant-derived GPPS to improve the efficiency of converting IPP and DMAPP to GPP, the result was not as expected. Therefore, further research can focus on the supply of the precursor GPP. Besides that, placing the entire biosynthetic pathway that utilizes the IUP pathway to produce linalool in different cellular compartments of yeast may further increase linalool production [21, 29].

## Methods

### Strains and culture media

*E. coli Trans*1-T1 phage resistant chemically competent cell used for the construction of plasmids was cultivated in Luria–Bertani (LB) medium with 50 μg/mL kanamycin at 37 °C. *S. cerevisiae* BY4742 derived from S288c was used as the parent strain and cultured at 30 °C in YPD medium (1% yeast extract, 2% peptone, 2% glucose). YPG medium (1% yeast extract, 2% peptone, 2% galactose), and YPS medium (1% yeast extract, 2% peptone, 2% Sucrose) were used to cultivate recombinant *S. cerevisiae* strains. SS-URA medium (synthetic complete drop-out medium with 2% Sucrose and without uracil) was used to cultivate engineered *S. cerevisiae* strains containing episomal plasmids. Synthetic complete drop-out medium without a specific component (SD-URA or SD-LEU) was used for auxotroph selection of *S. cerevisiae* transformants.SD-FOA (1 mg/mL of 5-fluoroorotic acid in synthetic complete drop-out medium) was used for screening *S. cerevisiae* strains with *KanMX-URA-PRB322ori* marker excision [53].

### Plasmids and strains construction

The relevant descriptions of the *S. cerevisiae* strains are summarized in Table 1. The corresponding plasmids and primers used in the study are listed in Additional file 1: Table S1 and S2, respectively. The pUMRI plasmids and the recombinant strains were constructed as described by Lv et al. [53]. The pUMRI-G, pUMRI-H, pUMRI-D, pUMRI-L, pUMRI-M, pUMRI-062, pUMRI-064 and pUMRI-R plasmids were constructed by replacing the *Ty4* homologous arm in plasmid pUMRI-16 [54] with *HO*, *DPP1*, *LPP1*, *MLS1*, *YPL062W*, *YJL064W* and *ROX1* homologous arms from *S. cerevisiae* BY4742, respectively. The *HO* homologous arms were amplified using primers HO-UP-F/HO-UP-R and HO-DN-F/HO-DN-R. The *DPP1* homologous arms were amplified using primers DPP1-UP-F/DPP1-UP-R and DPP1-DN-F/DPP1-DN-R. The *LPP1* homologous arms were amplified using primers LPP1-UP-F/LPP1-UP-R and LPP1-DN-F/LPP1-DN-R. The *MLS1* homologous arms were amplified using primers MLS1-UP-F/MLS1-UP-R and MLS1-DN-F/MLS1-DN-R. The *YPL062W* homologous arms were amplified using primers 62-UP-F/62-UP-R and 62-DN-F/62-DN-R. The *YJL064W* homologous arms were amplified using primers 64-UP-F/64-UP-R and 64-DN-F/64-DN-R. The *ROX1* homologous arms were amplified using primers ROX1-UP-F/ROX1-UP-R and ROX1-DN-F/ROX1-DN-R. The homologous arms above were individually introduced into pUMRI-16 linearized by primers pUMRI-F/pUMRI-R to generate the corresponding pUMRI vectors.

**Table 1:** Strains used in this study

Name	Description	Source
Strains		
BY4742	*MATα, his3Δ1, leu2Δ0, lys2Δ0, ura3Δ0*	[55]
BY4742-MC-02	BY4742, *ΔLPP1*:T_*CYC1*_-*ERG10*-*MLS*-P_*GAL1*_-P_*GAL10*_-*MLS*- *HMGS*-T_*ADH1*_; Δ*HO*: T_*TPS1*_-*tHMG1*-*MLS*-P_*GAL7*_-P_*GAL2*_-*MLS-ERG12*-T_*PGK1*_;	[21]
	Δ*DPP1*:T_*CYC1*_-*tHMG1-MLS*-P_*GAL1*_-P_*GAL10*_-*MLS-PMK*-T_*ADH1*_;	
	Δ*GAL80:*T_*TPS1*_-MVD1-MLS-P_*GAL7*_-P_*GAL2*_-*MLS-IDI1*-T_*PGK1*_	
	ΔP_*ERG20*_-*ERG20*::P_*HXT1*_-*ERG20*-P_*TEF1*_-*tHMG1*	
	Δ*TY4*:P_*GAL1*_-*IDI1*-T_*CYC1*_	
YMC214	BY4742-MC-02*,* pYC-*MLIS/MERG20*^*F96W–N127W*^	[21]
YMC217	BY4742-MC-02, pYC-*MAaLS1/MERG20*^*F96W−N127W*^	This study
YMC218	BY4742-MC-02, pYC-*Mt26AaLS1/MERG20*^*F96W−N127W*^	This study
YMC219	BY4742-MC-02, pYC-*Mt78AaLS1/MERG20*^*F96W−N127W*^	This study
Sclin01	BY4742, Δ*GAL80*::*LEU*2	This study
Sclin02	BY4742, Δ*GAL80*:: T_*CYC1*_-*AtIPK*-P_*GAL1*_-P_*GAL10*_-*ScCK*-T_*ADH1*_	This study
Sclin03	Sclin02, Δ*HO*::T_*CYC1*_-*t26AaLS1*-P_*GAL1*_-P_*GAL10*_-*ERG20*^*F96W−N127W*^-T_*ADH1*_	This study
Sclin04	Sclin03, Δ*Ty4*::T_*CYC1*_-*IDI1*-P_*GAL1*_-P_*GAL10*_-*IDI1*-T_*ADH1*_	This study
Sclin05	Sclin03, Δ*Ty4*::T_*CYC1*_-*IDI1*-P_*GAL1*_-P_*GAL10*_-*ERG20*^*F96W−N127W*^-T_*ADH1*_	This study
Sclin05-2	Sclin05, Δ*LPP1*::T_*CYC1*_-*t26AaLS1*-P_*GAL1*_-P_*GAL10*_-*ERG20*^*F96W−N127W*^-T_*ADH1*_	This study
Sclin05-3	Sclin05-2, Δ*DPP1*::T_*CYC1*_-*t26AaLS1*-P_*GAL1*_-P_*GAL10*_-*ERG20*^*F96W−N127W*^-T_*ADH1*;_	This study
Sclin05-5	Sclin05-3, Δ*MLS1*::T_*CYC1*_-*t26AaLS1*-P_*GAL1*_-P_*GAL10*_-*ERG20*^*F96W−N127W*^-T_*ADH1*;_	This study
	Δ*YPL062W*::T_*CYC1*_-*t26AaLS1*-P_*GAL1*_-P_*GAL10*_-*ERG20*^*F96W−N127W*^-T_*ADH1*_	
Sclin05-7	Sclin05-5, Δ*YJL064W*::T_*CYC1*_-*t26AaLS1*-P_*GAL1*_-P_*GAL10*_-*ERG20*^*F96W−N127W*^-T_*ADH1*;_ Δ*ROX1*::T_*CYC1*_-*t26AaLS1*-P_*GAL1*_-P_*GAL10*_-*ERG20*^*F96W−N127W*^-T_*ADH1*_	This study
Sclin05-C2	Sclin05, Δ*LPP1*	This study
Sclin05-C3	Sclin05-C2, Δ*DPP1*	This study
Sclin05-C5	Sclin05-C3, Δ*YPL062W*, Δ*MLS1*	This study
Sclin05-C7	Sclin05-C5, Δ*YJL064W*, Δ*ROX1*	This study

The gene *AaLS1* (GenBank ID: ADD81294) from *Actinidia arguta*, *AtIPK* (GenBank ID: AAN12957) from *Arabidopsis thaliana, tAgGPPS* (GenBank ID: AAN01134) from *Abies grandis* and *ScCK* (GenBank ID: AAA34499) from *Saccharomyces cerevisiae* were codon-optimized and synthesized by Sangon Biotech (Shanghai, China). The *ERG20*^*F96W−N127W*^, *IDI1* gene, and *LEU* expression cassette were amplified from the plasmids constructed previously [21]. The N-terminal plastid targeting sequence truncation site of *AaLS1* was predicted by the ChloroP or RR-heuristic method [31]. The full-length sequence of *AaLS1* and the N-terminus truncated *t26AaLS1* and *t78AaLS1* were amplified using primers AaLS1-F/AaLS1-R, t26AaLS1-F/t26AaLS1-R and t78AaLS1-F/t78AaLS1-R, respectively. Then the genes mentioned above were cloned into the pYC-*MLIS-MERG20*^*F96W−N127W*^ linearized by primers pYC-F/pYC-R to obtain pYC-*MAaLS1-MERG20*^*F96W−N127W*^, pYC-*Mt26AaLS1-MERG20*^*F96W−N127W*^ and pYC-*Mt78AaLS1-MERG20*^*F96W−N127W*^, respectively. *ERG20*^*F96W−N127W*^ and *IDI1* genes were amplified using primers E20-F/E20-R and IDI1-F/IDI1-R, respectively. The purified *t26AaLS1* and *ERG20*^*F96W−N127W*^ fragments were introduced into pUMRI-H/D/L/M/062/064/R using *Bam*H I/*Sal* I and *Eco*R I/*Spe* I, respectively, resulting in pUMRI-H/D/L/M/062/064/R-*t26AaLS1*-*ERG20*^*F96W−N127W*^. The purified *IDI1* and *ERG20*^*F96W−N127W*^ fragments were introduced into pUMRI-16-*IDI1* using *Eco*R I/*Spe* I to construct plasmids pUMRI-16-*IDI1*-*IDI1* and pUMRI-16-*IDI1*-*ERG20*^*F96W−N127W*^, respectively. The genes *AtIPK* and *ScCK* were amplified using primers AtIPK-F/AtIPK-R and ScCK-F/ScCK-R, respectively. The purified *AtIPK* and *ScCK* fragments were introduced into pUMRI-G using BamH I/Sal I and *Eco*R I/Spe I, respectively, resulting in plasmid pUMRI-G-*AtIPK*-*ScCK*. The *GAL80UP*-*LEU*-*Gal80DN* fragment was amplified using primers GAL80UP-F/GAL80UP-R, GAL80DN-F/GAL80DN-R, and LEU-F/LEU-R.

The plasmids or *Sfi* I-digested pUMRI plasmids were introduced into *S. cerevisiae* by electrotransformation performed with a Scientz-2C electroporation apparatus (Scientz Biotech, Ningbo, China). The *GAL80UP*-*LEU*-*GAL80DN* fragment was transformed into BY4742 and selected on SD-LEU plates to create strain Sclin01. To introduce the IUP into *S. cerevisiae*, the pUMRI-G-*AtIPK*-*ScCK* plasmid was transformed into BY4742 and selected on SD-URA plates to create strain Sclin02. The genes *t26AaLS1* and *ERG20*^*F96W−N127W*^ necessary for linalool production were integrated into the genome of Sclin02 using the recombinant pUMRI-H plasmid to obtain ScLin03. Similarly, the pUMRI-16-*IDI1*-*IDI1* and pUMRI-16-*IDI1*-*ERG20*^*F96W−N127W*^ plasmids were transformed into Sclin03 to create Sclin04 and Sclin05, respectively. The strains Sclin05-2/-3/-5/-7 were constructed using the same method above. Moreover, the recombinant pUMRI vectors were transformed into Sclin05 to create Sclin05-C2/C3/C5/C7. The genotypes of the recombinant strains were identified by PCR using primer pairs designed on the chromosomes and fragments at the integrated gene locations, respectively.

### Cultivation in shaking flasks

A single colony of the recombinant yeast was inoculated into 5 mL of YPD medium for overnight growth at 30 °C, 200 rpm. Then the precultures were inoculated into 20 mL of YPD/YPG/YPS at an initial OD_600_ of 0.05. The optimal combination of 17.5 mM isoprenol and isoprenol (in DMSO) was added to the culture medium as substrates supply for IPP and DMAPP. 20% isopropyl myristate was added to the medium to reduce isoprenol and/or prenol volatilization and to extract linalool. Engineered *S. cerevisiae* strains containing episomal plasmids were cultured overnight in 5 mL SD-URA medium at 30 °C, 200 rpm. Then, the precultures were transfered into 20 mL of SS-URA medium with 20% isopropyl myristate (IPM) at an initial OD_600_ of 0.05, and cultured for 3–4 days.

Analytical method The dry cell weight (DCW) of *S. cerevisiae* was determined by harvesting 2 mL wet cell culture, washing twice with water, centrifugation at 12,000 rpm for 5 min, and then measuring the weight of cell pellet dried at 105 °C for 36 h.

The linalool from isopropyl myristate supernatants was centrifuged at 12,000 rpm for 5 min, diluted in hexane, and analyzed by gas chromatography-mass spectrometry (GC-MS, Agilent 6890 N GC coupled with 59,751 MSD) equipped with the DB-5 MS column (30 m × 0.25 mm, 0.25 μm film thickness, Agilent, USA). The sample (1 μL) with naphthalene as internal standard was injected in splitless mode. The temperature of the injector and mass spectrometer source was 240 °C and 230 °C, respectively. The carrier gas was helium at a flow rate of 1 mL/min. The oven temperature was held at 50 °C for 1 min, subsequently increased to 115 °C at a rate of 5 °C/min and further raised to 300 °C at the rate of 10 °C/min (hold for 5 min). The detailed detection method of intracellular IPP/DMAPP was described in the previous study [28].

### Effects of isoprenol, prenol, and linalool on growth

The effects of isoprenol, prenol and linalool on the growth of *S. cerevisiae* strains were evaluated by measuring OD_600_. The frozen cells of strain BY4742 with 40 percent (vol/vol) glycerol were inoculated into YPD medium for overnight at 30 °C, 200 rpm. Then the cultures were transferred to YPS medium with different concentrations of isoprenol and/or prenol, and cultured for 24 h to make OD_600_ measurements. Similar operations were performed for the inhibitory effect of linalool on strain By4742-MC-02, recording the cell growth curve for 72 h.

## Supplementary Information

**Additional file 1: Fig. S1.** Inhibitory effects of linalool on strain BY4742-MC-02. Strain BY4742-MC-02 was grown in SS-URA medium with 0.5 % Tween 80 (v v^-1^) and cultured at 30°C, 200 rpm for 72 h. Then different concentrations of linalool were added to the culture and recorded the cell growth curve. All values represent the mean ± standard deviation from three biological replicates. **Fig. S2**. Effects of different combinations of isoprenol and prenol on *S. cerevisiae *growth. (A) Isoprenol was added to the YPD medium of strain BY4742 to a final concentration of 10, 25, 35, 50, 100 and 200 mM. (B) Prenol was added to the YPD medium of strain BY4742 to a final concentration of 10, 25, 35, 50, 100 and 200 mM. (C) Isoprenol and prenol were together added to the YPD medium of strain BY4742 to a final concentration of 10, 25, 35, 50, 100 and 200 mM. All values represent the mean ± standard deviation from three biological replicates. **Table S1.** Plasmids used in this study. **Table S2.** Primers used in this study.

## Data Availability

All data generated or analyzed during this study are included in this published article and its additional information file.
