# Supplementary material for: Enhancement of linalool production in Saccharomyces cerevisiae by utilizing isopentenol utilization pathway
Source: Microb Cell Fact. 2022 Oct 15;21:212. doi: 10.1186/s12934-022-01934-x (PMC9571491; doi:10.1186/s12934-022-01934-x)
Supplement: Supplementary file 1 — Additional file 1: Fig. S1. Inhibitory effects of linalool on strain BY4742-MC-02. Strain BY4742-MC-02 was grown in SS-URA medium with 0.5 % Tween 80 (v v-1) and cultured at 30°C, 200 rpm for 72 h. Then different concentrations of linalool were added to the culture and recorded the cell growth curve. All values represent the mean ± standard deviation from three biological replicates. Fig. S2. Effects of different combinations of isoprenol and prenol on S. cerevisiae growth. (A) Isoprenol was added to the YPD medium of strain BY4742 to a final concentration of 10, 25, 35, 50, 100 and 200 mM. (B) Prenol was added to the YPD medium of strain BY4742 to a final concentration of 10, 25, 35, 50, 100 and 200 mM. (C) Isoprenol and prenol were together added to the YPD medium of strain BY4742 to a final concentration of 10, 25, 35, 50, 100 and 200 mM. All values represent the mean ± standard deviation from three biological replicates. Table S1. Plasmids used in this study. Table S2. Primers used in this study. [file 12934_2022_1934_MOESM1_ESM.docx]

**Additional file 1**

**Fig. S1** **Inhibitory effects of linalool on strain BY4742-MC-02.** Strain BY4742-MC-02 was grown in SS-URA medium with 0.5 % Tween 80 (v v^-1^) and cultured at 30°C, 200 rpm for 72 h. Then different concentrations of linalool were added to the culture and recorded the cell growth curve. All values represent the mean ± standard deviation from three biological replicates.

**Fig. S2** **Effects of different combinations of isoprenol and prenol on *S. cerevisiae* growth.** (A) Isoprenol was added to the YPD medium of strain BY4742 to a final concentration of 10, 25, 35, 50, 100 and 200 mM. (B) Prenol was added to the YPD medium of strain BY4742 to a final concentration of 10, 25, 35, 50, 100 and 200 mM. (C) Isoprenol and prenol were together added to the YPD medium of strain BY4742 to a final concentration of 10, 25, 35, 50, 100 and 200 mM. All values represent the mean ± standard deviation from three biological replicates.

**Table S1** Plasmids used in this Study

| **Name** | **Description** | **Source** |
| --- | --- | --- |
| pESC-URA | 2*μ* *URA3*, T*_CYC1_*-*MCS2*-P*_GAL1_*-P*_GAL10_*-*MCS1*-T*_ADH1_* | Stored in the lab |
| pYC-*MAaLS1-MERG20^F96W-N127W^* | 2*μ* *URA3*, T*_CYC1_*-*AaLS*1-P*_GAL1_*-P*_GAL10_*-*ERG20^F96W-N127W^*-T*_ADH1_* | This study |
| pYC-*Mt26AaLS1-MERG20^F96W-N127W^* | 2*μ* *URA3*, T*_CYC1_*-*t26AaLS*1-P*_GAL1_*-P*_GAL10_*-*ERG20^F96W-N127W^*-T*_ADH1_* | This study |
| pYc-*Mt78AaLS1-MERG20^F96W-N127W^* | 2*μ* *URA3*, T*_CYC1_*-*t78AaLS*1-P*_GAL1_*-P*_GAL10_*-*ERG20^F96W-N127W^*-T*_ADH1_* | This study |
| pUMRI-16 | *loxp-KanMX-URA3-pbr322ori-loxp*,  T*_CYC1_*-*MCS2*-P*_GAL1_*-P*_GAL10_*-*MCS1*-T*_ADH1_*, *Ty4* homologous arm | ^1^ |
| pUMRI-H | *loxp-KanMX-URA3-pbr322ori-loxp*,  T*_CYC1_*-*MCS2*-P*_GAL1_*-P*_GAL10_*-*MCS1*-T*_ADH1_*, *HO* homologous arm | This study |
| pUMRI-D | *loxp-KanMX-URA3-pbr322ori-loxp*,  T*_CYC1_*-*MCS2*-P*_GAL1_*-P*_GAL10_*-*MCS1*-T*_ADH1_*, *DPP1* homologous arm | This study |
| pUMRI-L | *loxp-KanMX-URA3-pbr322ori-loxp*,  T*_CYC1_*-*MCS2*-P*_GAL1_*-P*_GAL10_*-*MCS1*-T*_ADH1_*, *LPP1* homologous arm | This study |
| pUMRI-G | *loxp-KanMX-URA3-pbr322ori-loxp*,  T*_CYC1_*-*MCS2*-P*_GAL1_*-P*_GAL10_*-*MCS1*-T*_ADH1_*, *GAL80* homologous arm | This study |
| pUMRI-M | *loxp-KanMX-URA3-pbr322ori-loxp*,  T*_CYC1_*-*MCS2*-P*_GAL1_*-P*_GAL10_*-*MCS1*-T*_ADH1_*, *MLS1* homologous arm | This study |
| pUMRI-062 | *loxp-KanMX-URA3-pbr322ori-loxp*,  T*_CYC1_*-*MCS2*-P*_GAL1_*-P*_GAL10_*-*MCS1*-T*_ADH1_*, *YPL062W* homologous arm | This study |
| pUMRI-064 | *loxp-KanMX-URA3-pbr322ori-loxp*,  T*_CYC1_*-*MCS2*-P*_GAL1_*-P*_GAL10_*-*MCS1*-T*_ADH1_*, *YJL064W* homologous arm | This study |
| pUMRI-R | *loxp-KanMX-URA3-pbr322ori-loxp*,  T*_CYC1_*-*MCS2*-P*_GAL1_*-P*_GAL10_*-*MCS1*-T*_ADH1_*, *ROX1* homologous arm | This study |
| pUMRI-16-*IDI1* | T*_CYC1_*-*IDI1*-P*_GAL1_*-P*_GAL10_*-*MCS1*-T*_ADH1_*, *Ty4* homologous arm | Stored in the lab |
| pUMRI-16-*IDI1-IDI1* | T*_CYC1_*-*IDI1*-P*_GAL1_*-P*_GAL10_*-*IDI1*-T*_ADH1_*, *Ty4* homologous arm | This study |
| pUMRI-16-*IDI1-ERG20^F96W-N127W^* | T*_CYC1_*-*IDI1*-P*_GAL1_*-P*_GAL10_*-*ERG20^F96W-N127W^*-T*_ADH1_*, *Ty4* homologous arm | This study |
| pUMRI-G-*AtIPK*-*ScCK* | T*_CYC1_*-*AtIPK*-P*_GAL1_*-P*_GAL10_*-*ScCK*-T*_ADH1_*, *GAL80* homologous arm | This study |
| pUMRI-H-*t26AaLS1*-*ERG20^F96W-N127W^* | T*_CYC1_*-*t26AaLS1*-P*_GAL1_*-P*_GAL10_*-*ERG20^F96W-N127W^*-T*_ADH1_*, *HO h*omologous arm | This study |
| pUMRI-D-*t26AaLS1*-*ERG20^F96W-N127W^* | T*_CYC1_*-*t26AaLS1*-P*_GAL1_*-P*_GAL10_*-*ERG20^F96W-N127W^*-T*_ADH1_*, *DPP1* homologous arm | This study |
| pUMRI-L-*t26AaLS1*-*ERG20^F96W-N127W^* | T*_CYC1_*-*t26AaLS1*-P*_GAL1_*-P*_GAL10_*-*ERG20^F96W-N127W^*-T*_ADH1_*, *LPP1* homologous arm | This study |
| pUMRI-M-*t26AaLS1*-*ERG20^F96W-N127W^* | T*_CYC1_*-*t26AaLS1*-P*_GAL1_*-P*_GAL10_*-*ERG20^F96W-N127W^*-T*_ADH1_*, *MLS1* homologous arm | This study |
| pUMRI-062-*t26AaLS1*-*ERG20^F96W-N127W^* | T*_CYC1_*-*t26AaLS1*-P*_GAL1_*-P*_GAL10_*-*ERG20^F96W-N127W^*-T*_ADH1_*, *YPL062W* homologous arm | This study |
| pUMRI-064-*t26AaLS1*-*ERG20^F96W-N127W^* | T*_CYC1_*-*t26AaLS1*-P*_GAL1_*-P*_GAL10_*-*ERG20^F96W-N127W^*-T*_ADH1_*, *YJL064W* homologous arm | This study |
| pUMRI-R-*t26AaLS1*-*ERG20^F96W-N127W^* | T*_CYC1_*-*t26AaLS1*-P*_GAL1_*-P*_GAL10_*-*ERG20^F96W-N127W^*-T*_ADH1_*, *ROX1* homologous arm | This study |

**Table S2** Primers used in this study

| **Primer name** | **Sequence (5′ to 3′)** |
| --- | --- |
| Primers used for strain construction | |
| GAL80-UP-F | ACTAAAGGGAACAAAAGCTGGAGCTGGCCTTGTGTAGTGCCCAATTGGGTGCCTCTATG |
| GAL80-UP-R | AGCACAGGGCAAGAGGCCTTTATGGCCCGAGAGTGCGCCGGTAAATGAAGAAAATATAG |
| GAL80-DN-F | ACCGGCGCACTCTCGGGCCATAAAGGCCTCTTGCCCTGTGCTTGGCCCCCAGTGCAG |
| GAL80-DN-F | CAGCTGGCGTAATAGCGAAGAGGCCTACAAGCGCAATTTACTAATGGCATTATATGG |
| HO-UP-F | ACTAAAGGGAACAAAAGCTGGAGCTGGCCTTCGAGATCACTTTTCGTGAT |
| HO-UP-R | TTTGATGCTGTCCGCGGGCCTCATGGGCCGAATCGCGTAAAAAGTTTGATT |
| HO-DN-F | ACTTTTTACGCGATTCGGCCCATGAGGCCCGCGGACAGCATCAAACTGTAA |
| HO-DN-R | TCAGCTGGCGTAATAGCGAAGAGGCCTACAGACGACCAGGTCAGCTAGGG |
| DPP1-UP-F | TCGGAAATCAACTTCTGTTCCATGTCGACTCACATTTCTTTGTATAAGATTG |
| DPP1-UP-R | GCAGTCACCCATCAGGCCTTTATGGCCGCATTATGTCCGATAAACACAGT |
| DPP1-DN-F | TTATCGGACATAATGCGGCCATAAAGGCCTGATGGGTGACTGCTTCCTCCA |
| DPP1-DN-R | TCAGCTGGCGTAATAGCGAAGAGGCCTACAGAAGGCTTGCCATTGGACAC |
| LPP1-UP-F | GAACAAAAGCTGGAGCTGGCCTTGTATGTATTGGTTGATAATTTTTGT |
| LPP1-UP-R | AGCCAAAATACCGATGGCCTTTATGGCCATGTTACTCAGTCCAACACTC |
| LPP1-DN-F | TGGACTGAGTAACATGGCCATAAAGGCCATCGGTATTTTGGCTTCGGTT |
| LPP1-DN-R | TGGCGTAATAGCGAAGAGGCCTACATGGAGCTGTTCTAGCATTTTTAG |
| MLS1-UP-F | AACAAAAGCTGGAGCTGGCCTTGTCAGTTTCAGGCAGGAAATCCAGATG |
| MLS1-UP-R | ATCTTATCGGCCTTTATGGCCTATCTATGAGCTTTGAATATAGTTTG |
| MLS1-DN-F | AAAGCTCATAGATAGGCCATAAAGGCCGATAAGATGATTCATTGCTAAC |
| MLS1-DN-R | TGGCGTAATAGCGAAGAGGCCTACATTCCAAATGGTGAGATTACTACCG |
| 62-UP-F | AACAAAAGCTGGAGCTGGCCTTGTAGTGTGTCTATTTGTCTGTTAGCG |
| 62-UP-R | ATACTATCAGAAGGCCTTTATGGCCCTCTTCTCATGTATCTACGTATC |
| 62-DN-F | TGAGAAGAGGGCCATAAAGGCCTTCTGATAGTATGTGTTTGTGTATG |
| 62-DN-R | GGCGTAATAGCGAAGAGGCCTACACGACAAAAGAAAAACGACCGAAA |
| 64-UP-F | TGAGTGGGTTATGGCCATAAAGGCCTTGGTGTTGGCATCATTGTCATC |
| 64-UP-R | TGGCGTAATAGCGAAGAGGCCTACAGATGACATCTCTGCCGCAATTGTG |
| 64-DN-F | ACAAAAGCTGGAGCTGGCCTTGTTCCTGACGCTATTCTCTCTTGTTTC |
| 64-DN-R | TGCCAACACCAAGGCCTTTATGGCCATAACCCACTCACATTGCAGAATC |
| ROX1-UP-F | ACAAAAGCTGGAGCTGGCCTTGTTGATTGTCTAACTGCGTTCTTTTGTG |
| ROX1-UP-R | AATGAGATATTGGCCTTTATGGCCTCTTTTGTTGTTCTTCCTCGTATTG |
| ROX1-DN-F | CAACAAAAGAGGCCATAAAGGCCAATATCTCATTGATTTGTTTTTTAA |
| ROX1-DN-R | TGGCGTAATAGCGAAGAGGCCTACAAACCACTGTCTCACACCAGGAAC |
| pUMRI-F | TGTAGGCCTCTTCGCTATTACGCCAGCTGAATTG |
| pUMRI-R | ACAAGGCCAGCTCCAGCTTTTGTTCCCTTTAG |
| AaLS1-F | TAACGTCAAGGAGAAAAAACCCCGGATCCATGGCTTCTTTCAACAGATTCTG |
| AaLS1-R | AAATCAACTTCTGTTCCATGTCGACCTAAGAAGAAGAGTCGTACAACATAG |
| T26AaLS1-F | ACGTCAAGGAGAAAAAACCCCGGATCCATGACTGCTGTTCCATCTATGCCAAC |
| T78AaLS1-F | TCAAGGAGAAAAAACCCCGGATCCATGAGAAGAGCTTTGAGAGAAGTTG |
| E20-F | AAGTAAGAATTTTTGAAAATTCGAATTCATGGCTTCAGAGAAGGAGATCCG |
| E20-R | TCGTCATCCTTGTAATCCATCGATACTAGTTTATTTACTTCTCTTGTATACCT |
| IDI1-F | AAGTAAGAATTTTTGAAAATTCGAATTCATGACCGCAGACAATAATAGCATG |
| IDI1-R | ATCCTTGTAATCCATCGATACTAGTTTACAGCATACGGTGAATCTGCCTG |
| AtIPK-F | CTTTAACGTCAAGGAGAAAAAACCCCGGATCCATGGAATTGAACATTTCTGA |
| AtIPK-R | AATCAACTTCTGTTCCATGTCGACTTACTTAGAGAATCTAATAATAGTACCC |
| ScCK-F | AAGTAAGAATTTTTGAAAATTCGAATTCAAATGGTTCAAGAATCTAGACCAG |
| ScCK-R | TCCTTGTAATCCATCGATACTAGTTTACAAGTAAGAAGTGTCCAAGAACTTG |
| GAL80UP-F | ATTGGGTGCCTCTATGATGGGTATGGCTTGGGCA |
| GAL80UP-R | GTGAAATACCGCACAGACGAGAGTGCGCCGGTAAATGA |
| GAL80DN-F | TGCACTCTCAGTACAATCTGAAGCATCTTGCCCTGTGCTTGGC |
| GAL80DN-R | CCATGCTACCTTCCATGGTTGAGCAAACCTATCACCCGGT |
| LEU-F | CTTCATTTACCGGCGCACTCTCGTCTGTGCGGTATTTCACACCGCATAT |
| LEU-R | GGGCCAAGCACAGGGCAAGATGCTTCAGATTGTACTGAGAGTGCACCAT |
| Primers used for genotype identification of recombinant strains | |
| GAL80-TEST-F | TTCCATGACGATCATCGTAGTGCCCAATTGGGT |
| LEU-TEST-R | TTTCAGAGGTCGCCTGACGCATATACCT |
| LEU-TEST-F | CATTAATATTGACAAGGAGGAGGGCA |
| GAL80-TEST-R | CAATTTACTAATGGCATTATATGGCCGAT |
| HOUP-TEST-F | ACCTCAACAGTAATTAACCCAAAG |
| Ori-R | TGTAGGTATCTCAGTTCGGTGTAGGTC |
| ScCK-TEST-F | TCTTTGACTTCTCAATCTTCTTCTTTG |
| HODN-TEST-R | ATATACACATTTTAGCAGATGCG |
| Ty4UP-TEST-F | GATACCTGGCAGTGACTCCTAG |
| IDI-TEST-F | AACGCCAAGGAGAACTTGACTG |
| Ty4DN-TEST-R | AAGGTAATGCATCATTCTATACGTG |
| DPP1UP-TEST-F | TATTTCGTATGTCATGTGGAG |
| E20-TEST-F | AGCAAATAGGAAAGATCGGAACTG |
| DPP1DN-TEST-R | AGCTCTTCATAAAGGGACAACAC |
| LPP1UP-TEST-F | TCAACCAACCCTTATAAACTTATATG |
| LPP1DN-TEST-R | ATACGCCTCAAGACGACAGATGACTAC |
| MLS1UP-TEST-F | ATTCTGTCATGAGTATTACTTATG |
| MLS1DN-TEST-R | TTGCTATACTTTCTCGACTTC |
| 062UP-TEST-F | ATTTACGCTGAAAGACTATGTTGAAG |
| 062DN-TEST-R | TCTTGAGCTTTCATAAACTTGTTG |
| 064UP-TEST-F | TTCATCAATATCCCTTAACTTTGGAAAG |
| 064DN-TEST-R | ATGTGGCTGTTAGTCAAGTCAG |
| ROX1UP-TEST-F | TTACTATGCAAAACAATTGGAAATCTG |
| ROX1DN-TEST-R | ACATTGTCAAATTCTTCAGGATTTCG |

1. Zhou, P. P.; Xie, W. P.; Li, A. P.; Wang, F.; Yao, Z.; Bian, Q.; Zhu, Y. Q.; Yu, H. W.; Ye, L. D., Alleviation of metabolic bottleneck by combinatorial engineering enhanced astaxanthin synthesis in Saccharomyces cerevisiae. *Enzyme and Microbial Technology* **2017,** *100*, 28-36.
